# Experimental Characteristics of Dry Stack Masonry under Compression and Shear Loading

**DOI:** 10.3390/ma8125489

**Published:** 2015-12-12

**Authors:** Kun Lin, Yuri Zarevich Totoev, Hongjun Liu, Chunli Wei

**Affiliations:** 1 Shenzhen Engineering Lab for Wind Environment and Technology, Shenzhen Key Lab of Urban & Civil Engineering Disaster Prevention & Reduction, Shenzhen Graduate School, Harbin Institute of Technology, Shenzhen 518055, China; linkun.hit@gmail.com; 2 Centre for Infrastructure Performance and Reliability, The University of Newcastle, University Drive, Callaghan, NSW 2308, Australia; Yuri.Totoev@newcastle.edu.au; 3 Bridge and Tunnel Division, Parsons Corporation, New York, NY 10005, USA; chunli.wei@parsons.com

**Keywords:** dry stack masonry, uniaxial compression behavior, shear-compression behavior, Mohr-Coulomb criterion, experiment

## Abstract

The behavior of dry stack masonry (DSM) is influenced by the interaction of the infill with the frame (especially the joints between bricks), which requires further research. This study investigates the compression and shear behaviors of DSM. First, a series of compression tests were carried out on both masonry prism with mortar (MP_m) and DSM prism (MP_ds). The failure mode of each prism was determined. Different from the MP_m, the stress-strain relationship of the MP_ds was characterized by an upward concavity at the initial stage. The compression strength of the MP_ds was slightly reduced by 15%, while the elastic modulus was reduced by over 62%. In addition, 36 shear-compression tests were carried out under cyclic loads to emphasize the influence of various loads on the shear-compression behavior of DSM. The results showed that the Mohr-Coulomb friction law adequately represents the failure of dry joints at moderate stress levels, and the varying friction coefficients under different load amplitudes cannot be neglected. The experimental setup and results are valuable for further research.

## 1. Introduction

Masonry is widely used worldwide because it is economical and easy to construct. However, the use of masonry is limited in seismic regions because of the inadequate tensile strength and the brittle behavior of the structure. To achieve good seismic performance, masonry is paired with strong materials designed to resist tension. One such dual-structural system is the reinforced concrete (RC) frame structure with masonry panels. RC frame structures with masonry infill panels are very common worldwide because they are practical and economical. However, the seismic performance of these structures, especially in high seismic regions, is disputable.

During an earthquake, both masonry and panel structures have a slight dissipation of energy in their elastic stage. Fracture failure of bricks and frames is the main cause of energy dissipation. Additionally, a large number of cross-shaped cracks on panels are difficult to repair [1,2]. To improve energy dissipation, Totoev and Lin [3] have directed their attentions to dry stack masonry (DSM) infilled reinforced concrete (RC) frame. The most obvious characteristic of DSM is its mortar-less build method. The structural behavior of DSM is not dependent on the performance of mortar and construction work technology, both of which are important factors that result in a high uncertainty of the traditional mortared masonry wall. Hence, DSM is a more stable structure than traditional mortared masonry. Furthermore, DSM is expected to improve efficiency, develop cyclic economy, and reduce environmental pollution [4,5].

A conceptually new system was proposed to increase the energy dissipation of DSM with framed mechanism [3]. According to this concept, masonry infill is considered as a distributed frictional damper. The panel with dry-stacked masonry units is capable of sliding relatively well in the plane of a wall, and is interlocked to prevent relative sliding out of the plane of the wall.

Previous research showed that the DSM in the new system could cause considerable energy dissipation to the RC frame [3], indicating that the behavior and mechanism of DSM panels contribute significantly to infilled RC frames. This study determines both the compressive properties (*i.e*., compressive strength, elastic modulus, and normal joint stiffness) and shear behavior (*i.e.*, cohesion, hysteretic loop, and variation of friction factor) of DSM by a series of experimental tests, such as uniaxial compression tests on dry mortar masonry prisms and cyclic shear tests on DSM joints.

## 2. Materials and Mechanics

Masonry is a combination of brick and mortar; the failure mechanism of masonry depends mainly on the behavior and interaction between each composition. The complex mechanical properties of masonry result from the following [6,7,8]: (1) brick and mortar are nonlinear materials with significant discrete characteristics; and (2) the tension and shear capability of mortar are much lower than brick, making mortar the weak link in the whole structure. Hence, the brick joints determine the entire mechanical characteristics of masonry.

### 2.1. Compression Behavior

Several experimental studies have been carried out on the compression behavior of masonry under different load cases [9,10,11,12,13,14,15,16], including diagonal-compression, flexural test, uniaxial compression test, cyclic compressive loading, and so on. According to the research, the stress-strain relationship has been achieved, the mechanical parameters, such as the elastic modulus yield strength for mortared masonry, have been determined. The influence of mortar strength, thickness, material of bricks, and composite systems on the compressive failure were also investigated experimentally and theoretically [17,18,19,20]. According to previous research, a series of mechanical models of masonry joints under compression have been proposed and corresponding simplified methodology has been provided for application in practical engineering. However, there are rare cases of research on the compression behavior of dry stack masonry. DSM is considered an extreme case compared with traditional masonry because of the close-to-zero thickness of mortar between bricks. Under this condition, compressive strength and elastic modulus are significantly affected by the connection between bricks. Interface behavior is affected by roughness, hardness of contact, and is characterized as a significant nonlinear behavior. Uniaxial compression experiments were carried out to investigate mortar influence under two different scenarios: Masonry prism built with mortar (MP_m) and dry stack masonry prism built without mortar (MP_ds).

**Figure 1:**
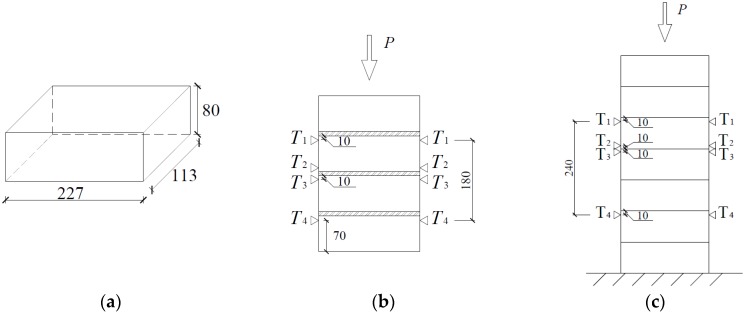
Sketch of brick specimen. (**a**) demission of brick; (**b**) positions of LVDTs in MP_m; and (**c**) positions of linear variable displacement transducers (LVDTs) in MP_ds. (units: mm).

Sandstone, a common solid concrete brick with dimensions of 227 mm × 113 mm × 80 mm, was used for testing (Figure 1a). The material behavior of the brick was determined from a material test. Lin [21] studied the brick mechanical properties in detail, and indicated a Young’s modulus of 26,365 N/mm^2^, a Poisson’s ratio of 0.3, and a density of 2250 kg/m^3^.

Eight prisms were constructed for MP_m. The first specimen (specimen 0) was used to confirm the compression strength for further testing. Compression specimens were constructed and tested according to the Australia Standard (AS3700) [22]. According to AS3700, there are must no less than 3 bricks for the testing prism, and the height/thickness ratio of prism should between 3 and 5. Compression tests of the four-brick-high masonry prisms determined the elastic properties of brick units and mortar joints, as well as the compressive strength of MP_m. Seven-brick-high masonry prisms, with a height and thickness ratio of greater than 5 (minimizing the influence of platen restraint), was used to achieve corresponding behaviors of MP_ds.

**Figure 2:**
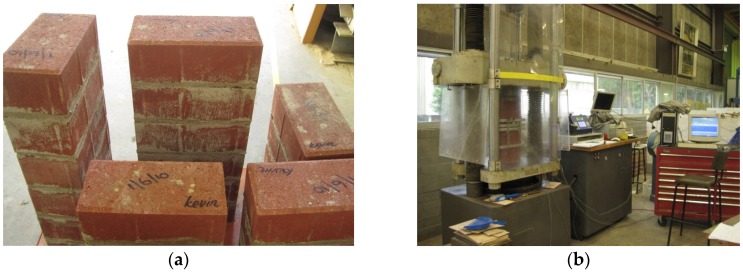
Photo of experimental instruments: (**a**) masonry prism built with mortar; (**b**) loading equipment.

**Figure 3:**
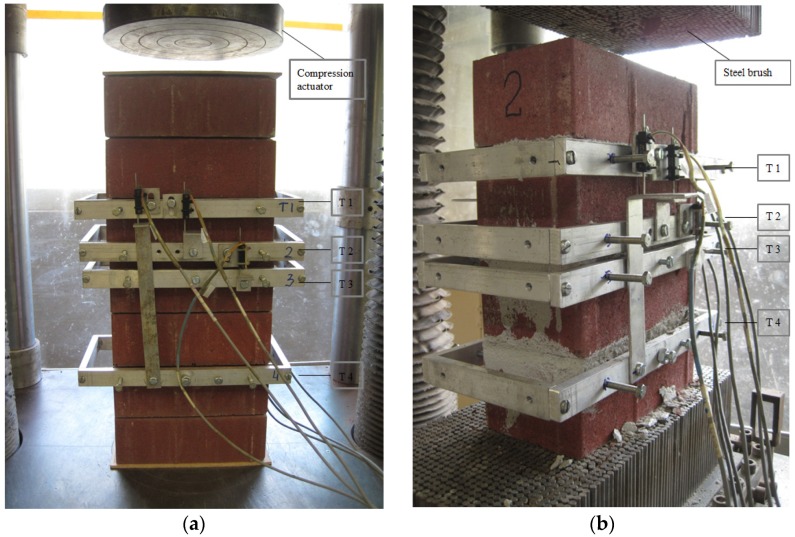
Set up of the experiments: (**a**) test setup of MP_m; (**b**) test setup of MP_ds.

Linear variable displacement transducers (LVDTs) were placed on both sides of the specimen to measure displacement across mortar joints and across three bricks, and thus, calculate strain of mortar joint and masonry. LVDTs were not used to measure the displacement within a single brick unit because they are not sensitive enough to measure small brick displacement. LVDTs were mounted onto brackets, which were screwed into the specimen at fine target points to accurately determine the gauge lengths for displacement measurement. The LVDT positions are shown in Figure 1b,c. The experimental instruments are shown in Figure 2. The masonry prism setups are shown in Figure 3a,b. The compressive load was divided by the average cross section to determine the compressive stress under each load case. The average value of vertical displacement recorded by the two LVDTs was chosen. The average vertical displacement divided by the gage length of LVDTs (the initial distance between two installation positions of LVDTs, illustrated in Figure 1b,c) is the corresponding strain ε.

The compression test was used to improve the determination of the elastic modulus. Each specimen was loaded and unloaded three times before finally being loaded into failure mode. Before unloading, specimens were loaded to approximately 40% of their predicted peak loads to capture the elastic loading range and minimize non-recoverable damage. Specimen 1 was loaded to 150 kN before unloading (based on an estimate of P_c_ = 375 kN), specimens 2 to 4 were loaded to 210 kN before unloading (40% of P_c_ specimen 1), and specimens 5 to 7 were loaded to 240 kN before unloading (approximately 40% of the average P_c_ of specimens 1 to 4). The average value of the displacements recorded from the second, third, and final load cycles were used to calculate the elastic modulus of the masonry and mortar. Displacements recorded from the first load cycle were ignored. All the compression tests were stopped upon reaching the ultimate load to avoid damaging the potentiometers.

### 2.2. Shear Behavior

The compression-shear behavior of mortared masonry joints has been deeply investigated for a number of masonry classes by, among others, Atkinson *et al*. [23], van der Pluijm [24], van Zijl [25], Lourenço *et al*. [26], Augenti and Parisi [27]. The shear displacement curves and failure modes of mortared masonry have been investigated. Additionally, series constitutive models, which are able to be used for engineering application, have been proposed. However, the previous traditional masonry joint studies, which were used as reference for the present study, cannot be used for DSM directly because research on shear behavior of DSM joints is limited.

**Figure 4:**
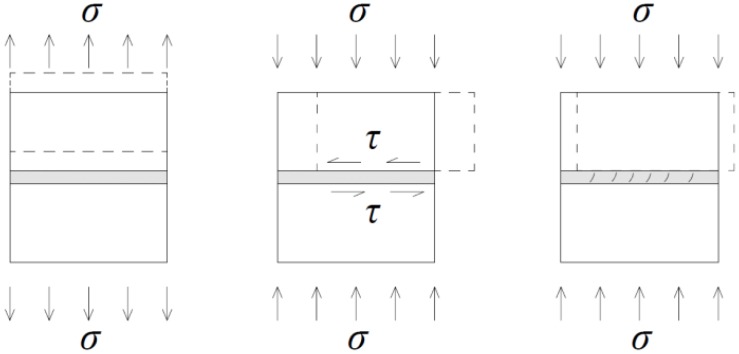
Failure mode of masonry joints.

**Figure 5:**
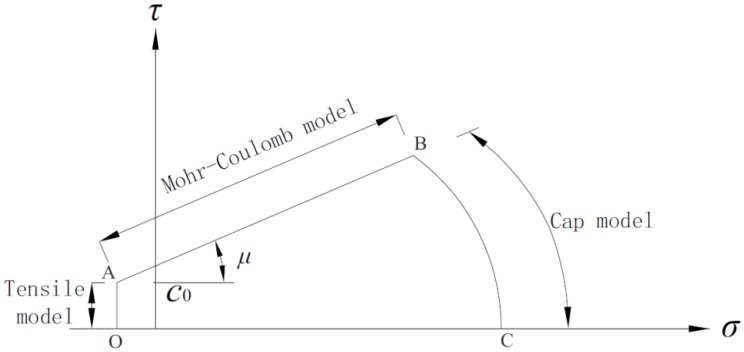
Mechanical mode of masonry joints.

The failure mode of mortar joints under different pre-compression levels is sorted into three types [28,29,30]: tensile damage, shear damage, and compression-shear damage (Figure 4). Cohesion and mortar tensile strength dominated tensile failure. A graphical representation of this failure type is presented in branch OA of Figure 5. Initial cohesion and shear friction factor dominated joint failures under medium pre-compression, which is described as the Mohr–Coulomb failure criterion (branch AB of Figure 5). A diagonal cracking in the mortar and the cap model occurred (branch BC of Figure 5) as normal compressive stresses increased. This occurrence represents the joint failure mode.

Coulomb friction law is suited to masonry joints under medium pre-compression, establishing a linear relationship between shear stress τ and normal stress σ, which is given as:

τ = c_0_ + μσ (1)

In the equation, c_0_ represents the cohesion. According to previous research [31], the c_0_ is assumed as zero in the case of dry masonry joints due to insignificant roughness at the interface. Additionally, μ represents the friction factor, which characterizes the contact surface. A series of tests was carried out to characterize the Mohr–Coulomb failure criterion. The influence of cyclic loading on the shear properties of masonry joints was investigated.

**Figure 6:**
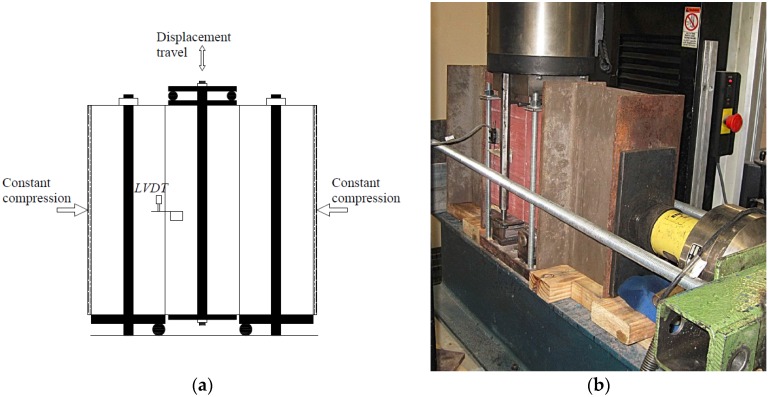
Compression-shear test of DSP. (**a**) schematic diagram of test; (**b**) photo of test.

The cyclic triplet test was set up based on various European standard triplet shear tests [32] (shown as Figure 6a). Two independent actuators were used in this test: one for vertical cyclic displacement and another for horizontal constant pressure. The embedded pressure sensor of the vertical actuator achieved real-time vertical shear load. The LVDTs positioned in the direction of the shear load measured the relative displacement (Figure 6b).

The definition of imposed horizontal displacement histograms was based on previous research [31]. This test used three pressure load cases: L series (0.1 MPa), M series (0.3 MPa), and H series (0.5 MPa). Three specimens were tested for each pressure load case. Four displacement amplitudes (with corresponding velocities) were used for each specimen under single pressure: ±0.8 mm (1 mm/min), ±1.6 mm (2 mm/min), ±2.4 mm (2 mm/min), and ±3.2 mm (4 mm/min).

## 3. Experimental Results and Discussion

### 3.1. Compression Test

**Figure 7:**
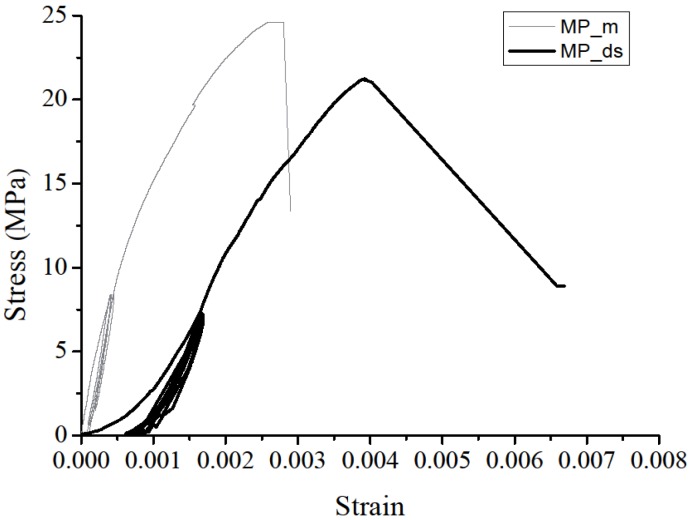
Typical normal stress-strain curve of different masonry prisms.

The typical strain-stress curve of masonry joints under compression is shown in Figure 7. The MP_m stress-strain diagram was characterized by a downward concavity, which was also reported by previous studies [33,34]. The MP_m under monotonic uniaxial compression test showed that the strain-stress curve demonstrated a linear behavior as the stress below 40% of compression strength, whereas the strain-stress curve demonstrated a nonlinear behavior as the stress above 70% of uniaxial strength (15 MPa).

The MP_ds strain-stress is divided into two parts: the pre-compression stage and the real-compression stage. The MP_ds stress-strain curves achieved a significant upward concavity because of the initial gap between bricks. The initial gap, however, started to close under compression stress, which tightened the connection between bricks, thus, increasing the prism elastic modulus. Similar results were also reported by other scholars [35].

**Figure 8:**
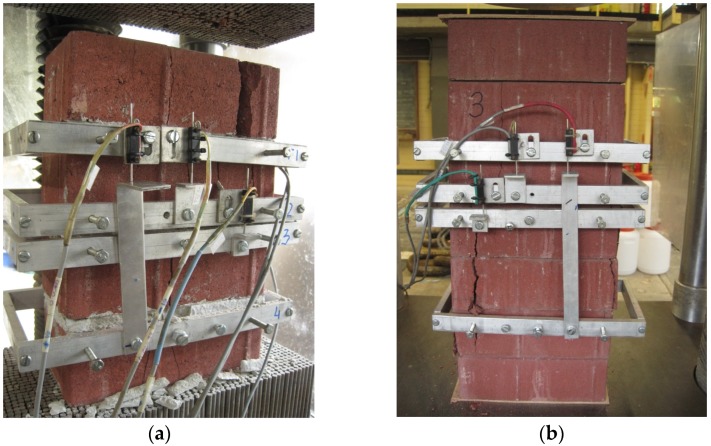
Typical failure modes of different masonry prisms: (**a**) MP_m; (**b**) MP_ds.

All specimens for the MP_m failed because of the mortar joint crushing and vertical split cracking through the front and back face of the brick units (Figure 8a). The lateral tensile stress in brick units caused this failure mode and extended through the height. Other scholars [17,25] reported similar experimental results.

A typical inward tensile crush/crack was observed for the MP_ds. The damage focused on the middle bricks, and cracks occurred at the prism edges (Figure 8b). This mode was mostly caused by: (1) stress concentrating on the middle of the brick prism, and (2) the crack not spreading across the brick because the dry stack joint acts as one of the most effective paths for stress release.

Table 1 shows the maximum compression load (P_c_), corresponding maximum compressive stress (f_c_), masonry strain at f_c_, and masonry elastic modulus (E_mas_). Masonry elastic modulus (E_mas_) was commonly calculated as the chord modulus of linear part of the masonry compression stress-strain curve, which is generally defined to be between 5% and 33% of the maximum compressive strength [6]. The compression strength of the MP_ds had a slight reduction (15%) compared with that of the MP_m, while the elastic modulus was reduced by more than 62%.

**Table 1:** Experimental result of uniaxial compression tests.

No.	Masonry with Mortar			Masonry without Mortar		
	Maximum Compression *P*_c_ (kN)	Compression Strength *f*_c_ (MPa)	Elastic Modulus *E* (MPa)	Maximum Compression *P*_c_ (kN)	Compression Strength *f*_c_ (MPa)	Elastic Modulus *E* (MPa)
1	524.3	20.4	15,380	456.6	17.8	7982.6
2	635.3	24.7	23,065	477.9	18.6	8851.3
3	662.9	25.8	22,067	473.5	18.4	6424.6
4	589.9	22.9	23,029	–	–	–
5	575.5	22.4	18,796	–	–	–
6	420.4	16.4	13,709	–	–	–
7	635.0	24.7	21,777	–	–	–
Mean value	551.7	21.5	20,407	469.3	18.3	7702.3
CV (%)	15.09	14.99	18.73	2.40	2.28	15.96

### 3.2. Shear Test

The results of compression-shear test are listed in Table 2, which include displacement amplitude (*D*), shear force (*F*), compression force (*N*), shear stress (τ), normal stress (σ), friction factor (μ), average friction factor under different displacement amplitudes (${\bar{\mu }}_{A}$), and average friction factor under different compression force (${\bar{\mu }}_{\sigma }$). The load cases are referred to as “L_1_2,” where “L” refers to the loading series, “1” refers to the number of specimens, and “2” refers to the applied displacement of second amplitudes (*i.e.*, 1.6 mm).

**Table 2:** Experimental results of shear-compression test.

Load Cases	D (mm)	F (kN)	N (kN)	τ (MPa)	σ (MPa)	μ	${\bar{\mu }}_{A}$	${\bar{\mu }}_{\sigma }$
L_1_1	0.80	2.93	2.70	0.06	0.11	0.54	0.51	0.48
L_1_2	1.60	2.65	2.57	0.05	0.10	0.52		
L_1_3	2.40	3.00	3.00	0.06	0.12	0.50		
L_1_4	3.20	2.55	2.55	0.05	0.10	0.50		
L_2_1	0.80	1.50	2.46	0.03	0.10	0.30	0.30	
L_2_2	1.60	1.18	2.51	0.02	0.10	0.23		
L_2_3	2.40	1.70	2.66	0.03	0.10	0.32		
L_2_4	3.20	1.70	2.54	0.03	0.10	0.34		
L_3_1	0.80	2.60	2.55	0.05	0.10	0.51	0.45	
L_3_2	1.60	2.55	2.65	0.05	0.10	0.48		
L_3_3	2.40	2.25	2.63	0.04	0.10	0.43		
L_3_4	3.20	2.00	2.55	0.04	0.10	0.39		
M_1_1	0.80	9.00	7.77	0.18	0.30	0.58	0.57	0.56
M_1_2	1.60	8.75	7.73	0.17	0.30	0.57		
M_1_3	2.40	9.10	7.87	0.18	0.31	0.58		
M_1_4	3.20	8.25	7.36	0.16	0.29	0.56		
M_2_1	0.80	8.70	7.72	0.17	0.30	0.56	0.55	
M_2_2	1.60	8.60	7.67	0.17	0.30	0.56		
M_2_3	2.40	8.50	7.77	0.17	0.30	0.55		
M_2_4	3.20	8.25	7.77	0.16	0.30	0.53		
M_3_1	0.80	8.48	7.65	0.17	0.30	0.55	0.55	
M_3_2	1.60	8.40	7.64	0.16	0.30	0.55		
M_3_3	2.40	8.28	7.60	0.16	0.30	0.54		
M_3_4	3.20	8.30	7.65	0.16	0.30	0.54		
H_1_2	1.60	16.00	12.60	0.31	0.49	0.64	0.62	0.62
H_1_3	2.40	16.00	12.82	0.31	0.50	0.62		
H_1_4	3.20	15.00	12.76	0.29	0.50	0.59		
H_2_1	0.80	18.00	12.94	0.35	0.50	0.70	0.66	
H_2_2	1.60	17.65	13.11	0.34	0.51	0.67		
H_2_3	2.40	17.00	13.08	0.33	0.51	0.65		
H_2_4	3.20	16.50	12.96	0.32	0.51	0.64		
H_3_1	0.80	15.25	12.85	0.30	0.50	0.59	0.59	
H_3_2	1.60	15.13	12.88	0.29	0.50	0.59		
H_3_3	2.40	15.00	12.84	0.29	0.50	0.58		
H_3_4	3.20	14.75	12.77	0.29	0.50	0.58		

#### 3.2.1. Hysteretic Behavior

**Figure 9:**
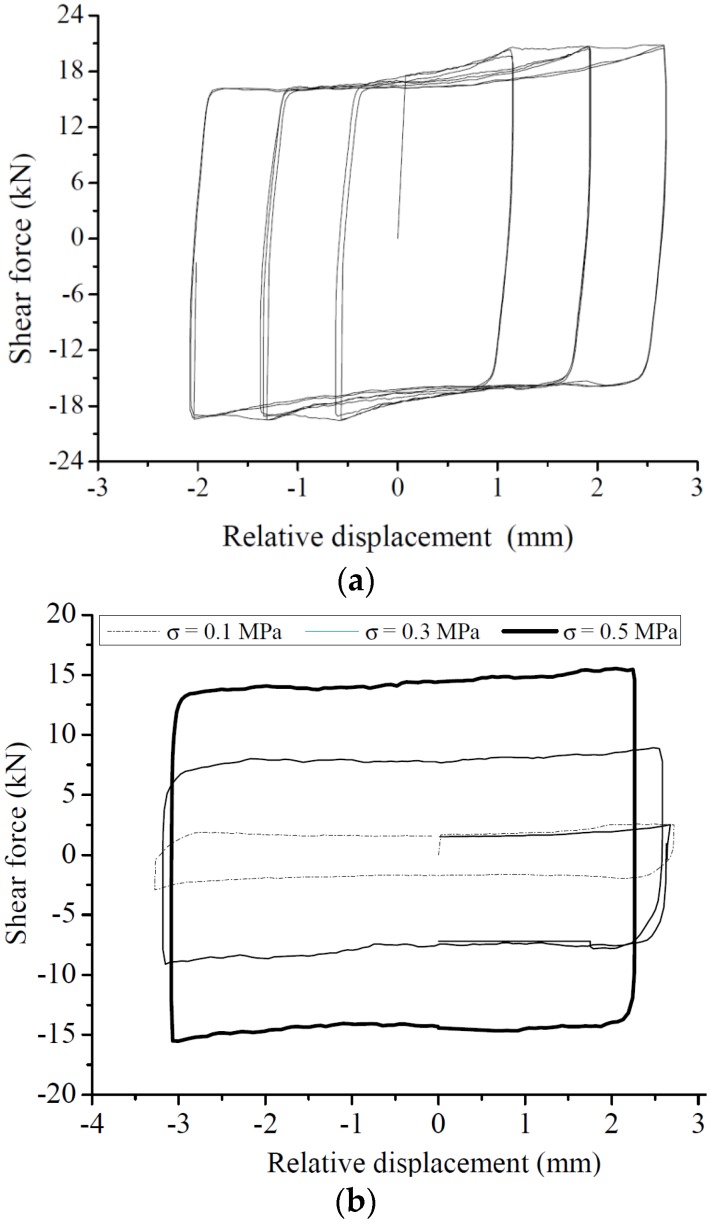
Typical hysteretic loop. (**a**) different amplitude with constant compression (σ = 0.5 MPa); (**b**) different compression with same amplitude.

**Figure 10:**
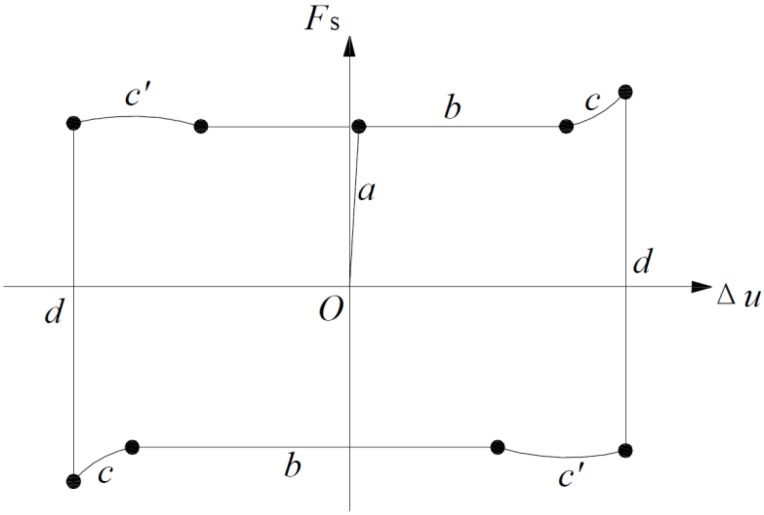
Mechanical model of the dry stack masonry joint.

**Figure 11:**
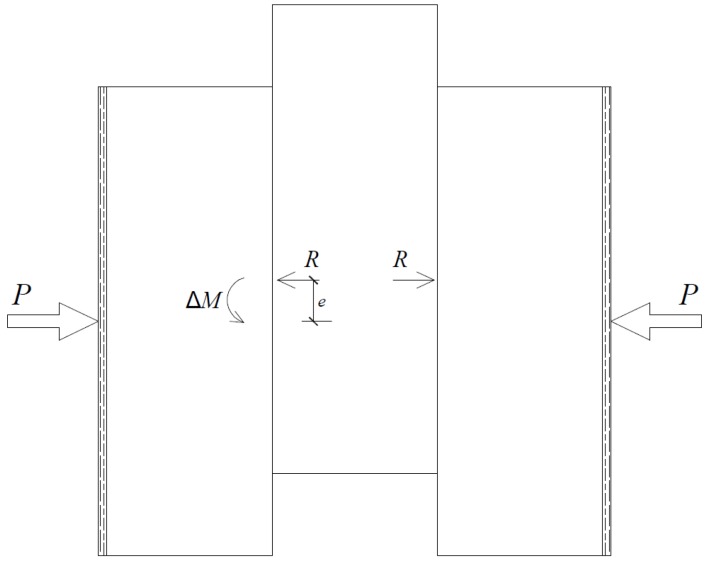
Schematic of additional bending moment.

Figure 9 shows a typical hysteretic loop, which is summarized as a mechanical model in Figure 10. The following behaviors are recognized:

(1) A significant elasto-plastic behavior is found. At the initial loading stage (Stage a), the force-displacement curve is linear. After the brick starts to slide, friction force is constant, thus, exhibiting a fully plastic behavior (Stage b).
(2) The load-displacement curve exhibits obvious “pinching” in the hysteretic graphs (Stage c and c’), which increases with increasing displacement. Considering the test set-up, “pinching” may occur because of the applied pre-compression force combined with the eccentricity in the reaction force when Unit B was moving toward maximum displacement. Figure 11 is a schematic of the occurring forces.
(3) Reload stage (Stage d) exhibits rigid behavior as pre-compression increases (Figure 10b).
(4) The imperfect connection between the actuator and the middle brick causes the asymmetric behavior of the hysteretic curve.

#### 3.2.2. Coulomb Failure Criteria and Shear Friction Degradation

**Figure 12:**
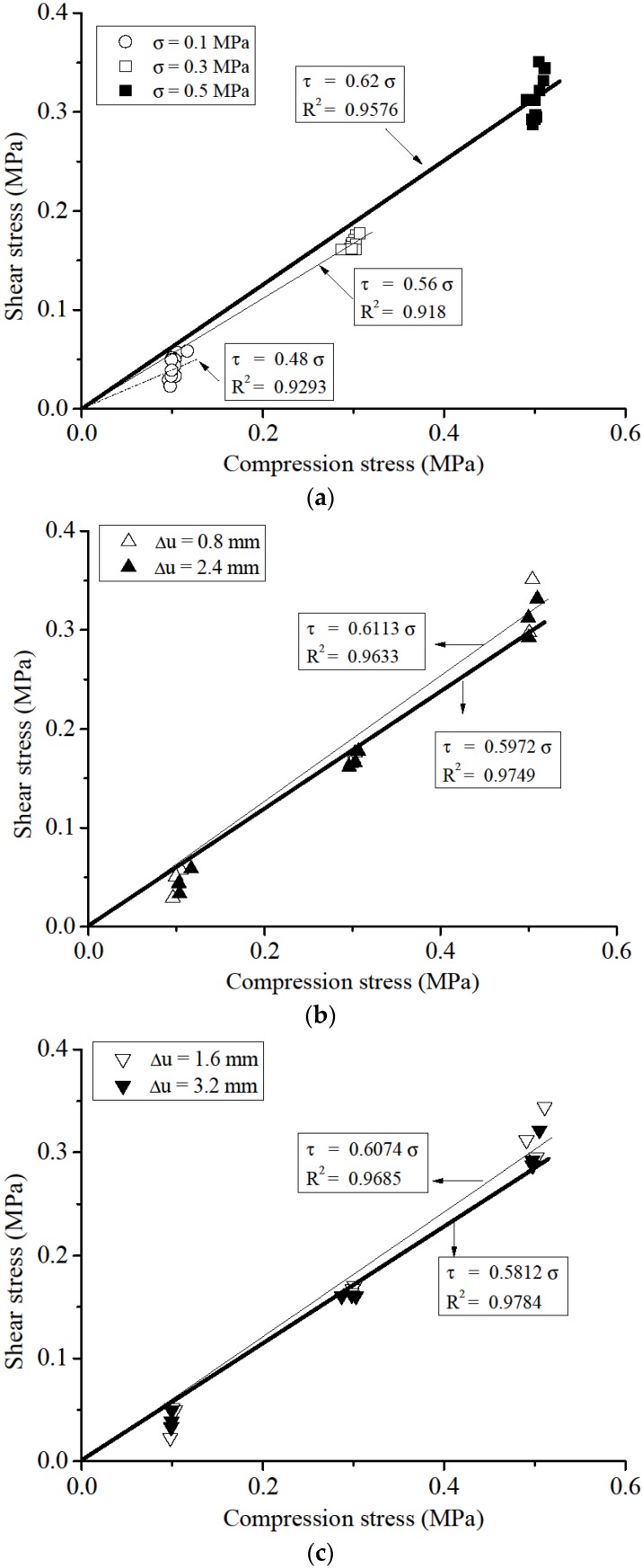
Experimental failure criteria: (**a**) different compression stress; (**b**) different amplitudes-1; and (**c**) different amplitudes-2.

**Figure 13:**
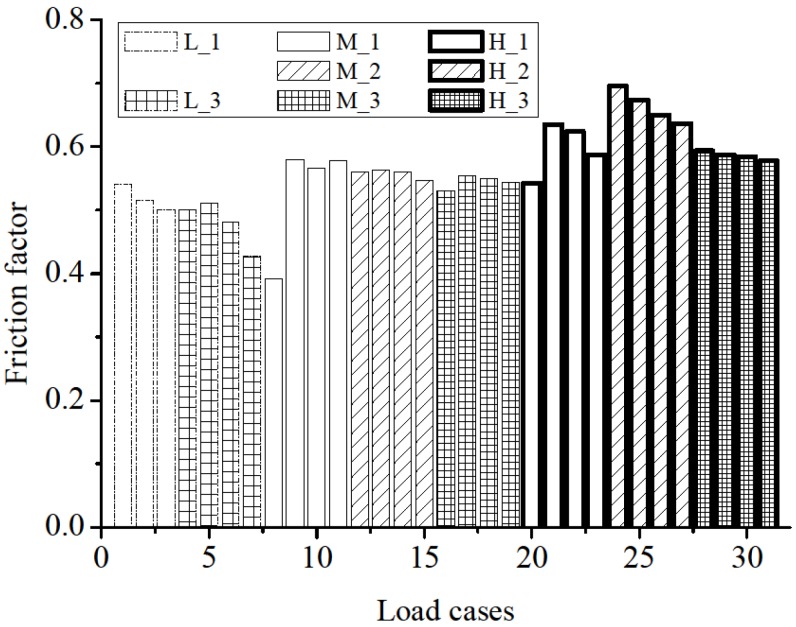
Friction factor decrease under different load cases.

**Figure 14:**
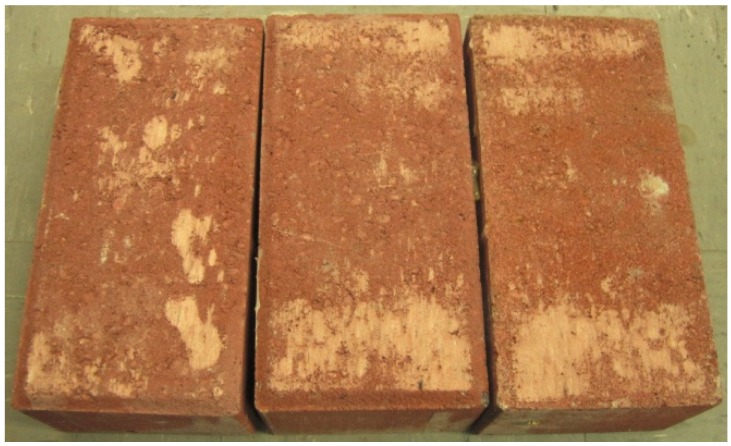
Wear surface after test.

Shear and compression stress are calculated by the average force achieved during the test:

(2) $$\begin{array}{c} \tau \text{=}\frac{F}{2A}\\ \sigma =\frac{N}{A} \end{array}$$

where F is the shear force that equals to the vertical applied force; N is the normal force that equals to the horizontal applied force; and A is the contact area in this experiment. Shear stress under different compressive stress and displacement amplitudes is shown in Figure 12 and Figure 13, respectively. The results of L_2 were disregarded because of their significant discrepancy. The figures show us that:

(1) The influence of normal stress to the frictional factor of the DSM can not be ignored. From Figure 12a, a considerable increase of 29% of the frictional factor is found when normal stress increases from 0.1 MPa to 0.5 MPa. Meanwhile, a more stabilized shear stress amplitude is found under higher normal stress level. This variation indicates the shear mechanical behaviour of DSM joints mainly depends on how closely two interfaces contact. For practical test, although there is no significant roughness at the interface, the micro burr exists. As the normal stress increases, the surface contact tighter and the micro burr interlocking with each other more firmly. Therefore, the resistance between bricks is enhanced, which results in the increase of frictional factor.
(2) The frictional factor and its discrepancy decreases as load amplitude increases under the same compression stress. The shear friction coefficient was 0.61 and the correlation coefficient was 0.96 for the initial condition (minimum displacement amplitude Δu = 0.8 mm). In contrast, the shear friction coefficient was 0.58 and the correlation coefficient was 0.98 for the final condition (maximum displacement amplitude Δu = 3.2 mm according to past investigations by van Zijl [25], and Augenti and Parisi [27]. The progressive wearing of blocks caused these coefficient conditions (Figure 14).

## 4. Conclusions and Suggestions

This paper presents the experimental results on the characterization of shear and uniaxial behavior of dry stack masonry joints under cyclic loading. Solid concrete brick, a material common in the AU, was used in all the tests.

The series-compression test examined both the compression strength and elastic modulus of the MP_ds and could lead to further research. Compared to MP_m stress-strain curves, the MP_ds stress-strain curves were characterized by an upward concavity at the initial stage. The compression strength had a slight reduction (15%), whereas the elastic modulus was reduced by over 62%. A typical inward tensile crush/crack was discovered. The damage was focused on the middle bricks and the cracks occurred at the prism edges.

Shear experiments were set up based on various European standard triplet shear tests. The cyclic displacement control was used. The horizontal confining pressure was kept constant while the test was carried out under vertical displacement control. Hysteretic loop curves were achieved and a significant pinch behavior caused by additional bending moment was found. For future research, additional bending moments should be avoided in cyclic shear-compression tests. The experimental results indicated that the Coulomb friction law adequately represents the failure of dry masonry joints. Friction factor was observed to increase along with pre-compression stress and to decrease along with applied amplitude. Large pre-compression and small displacement amplitude were observed to lead to a large friction factor.

## References

[1] Bayraktar A., Altunişik A.C., Pehlivan M. (2013). Performance and damages of reinforced concrete buildings during the October 23 and November 9, 2011 Van, Turkey, earthquakes. Soil Dyn. Earthq. Eng..

[2] Romão X., Costa A.A., Paupério E., Rodrigues H., Vicente R., Varum H., Costa A. (2013). Field observations and interpretation of the structural performance of constructions after the 11 May 2011 Lorca earthquake. Eng. Fail. Anal..

[3] Lin K., Totoev Y.Z., Liu H.J. Energy Dissipation During Cyclic Tests in Framed Dry Stack and Unreiforced Masonry Panels. Proceeding of the Ninth Australasian Masonry Conference.

[4] Uzoegbo H.C., Senthivel R., Ngowi J.V. (2007). Loading capacity of dry-stack masonry walls. Mason. Soc. J..

[5] Bansal D. Interlocking Dry Stacked Masonry. Proceeding of the Eight International Masonry Conference.

[6] Tomazevic M., Lutman M. (1996). Seismic behavior of masonry walls: Modeling of hysteretic rules. J. Struct. Eng..

[7] Lourenço P.B., Rots J.G., Blaauwendraad J. (1998). Continuum model for masonry: Parameter estimation and validation. J. Struct. Eng. ASCE.

[8] Lin K., Totoev Y.Z., Liu H.J., Page A.W. (2014). Modeling of dry-stacked masonry panel confined by reinforced concrete frame. Arch. Civ. Mech. Eng..

[9] Turnšek V., Čačovič F. Some Experimental Results on the Strength of Brick Masonry Walls. Proceeding of the Second International Brick Block Masonry Conference.

[10] Kent D.C., Park R. (1971). Flexural members with confined concrete. J. Struct. Div..

[11] Calvi G.M., Magenes G. Experimental Evaluation of Seismic Strength of Old Masonry Structures. Proceeding of Nineth International Brick Block Masonry Conference.

[12] Naraine K., Sinha S. (1989). Behavior of brick masonry under cyclic compressive loading. J. Struct. Eng..

[13] Augenti N., Parisi F. (2010). Constitutive models for tuff masonry under uniaxial compression. J. Mater. Civ. Eng..

[14] Parisi F., Augenti N. (2013). Assessment of unreinforced masonry cross sections under eccentric compression accounting for strain softening. Constr. Build. Mater..

[15] Li J., Masia M.J., Stewart M.G., Lawrence S.J. (2014). Spatial variability and stochastic strength prediction of unreinforced masonry walls in vertical bending. Eng. Struct..

[16] Zavalis R., Jonaitis B., Marčiukaitis G. (2013). Numerical and experimental analysis of grouted hollow block masonry under compression. Eng. Struct. Tech..

[17] Faella C., Martinelli E., Paciello S., Camorani G., Aiello M.A., Micelli G., Nigro E. (2011). Masonry columns confined by composite materials: Experimental investigation. Compos. Part B Eng..

[18] Faella C., Martinelli E., Camorani G., Aiello M.A., Micelli G., Nigro E. (2011). Masonry columns confined by composite materials: Design formulae. Compos. Part B Eng..

[19] Mazzotti C., Ferracuti B., Bellini A. (2015). Experimental bond tests on masonry panels strengthened by FRP. Compos. Part B Eng..

[20] Micelli F., Ludovico M.D., Balsamo A., Manfredi G. (2014). Mechanical behaviour of FRP-confined masonry by testing of full-scale columns. Mater. Struct..

[21] Lin K., Totoev Y.Z., Liu H.J. (2012). Quasi-static experimental research on dry-stack masonry infill panel frame. J. Build. Struct..

[22] (2001). AS 3700-2001, Australian Standard, Masonry Structures.

[23] Atkinson R.H., Amadei B.P., Saeb S., Sture S. (1989). Response of masonry bed joints in direct shear. J. Struct. Eng..

[24] Van der Pluijm R. Shear behaviour of bed joints. Proceedings of the Sixth North American Masonry Conference.

[25] Van Zijl G. (2004). Modeling masonry shear-compression: Role of dilatancy highlighted. J. Eng. Mech..

[26] Lourenço P.B., Barros J.O., Oliveira J.T. (2004). Shear testing of stack bonded masonry. Constr. Build. Mater..

[27] Augenti N., Parisi F. (2011). Constitutive modelling of tuff masonry in direct shear. Constr. Build. Mater..

[28] Lourenço P.B., Rots J.G. (1997). Multisurface interface model for analysis of masonry structures. J. Eng. Mech..

[29] El-Sakhawy N.R., Raof H.A., Gouhar A. (2002). Shearing behavior of joints in load-bearing masonry wall. J. Mater. Civ. Eng..

[30] Rahman A., Ueda T. (2014). Experimental investigation and numerical modeling of peak shear stress of brick masonry mortar joint under compression. J. Mater. Civ. Eng..

[31] Lourenço P.B., Ramos L.F. (2004). Characterization of cyclic behavior of dry masonry joints. J. Struct. Eng..

[32] European Commission for Standardization (2002). Methods of Test for Masonry.

[33] Almeida C., Paulo Guedes J., Arede A., Costa C.Q., Costa A. (2012). Physical characterization and compression tests of one leaf stone masonry walls. Constr. Build. Mater..

[34] Kaushik H.B., Rai D.C., Jain S.K. (2007). Stress-strain characteristics of clay brick masonry under uniaxial compression. J. Mater. Civ. Eng..

[35] Andreev K., Sinnema S., Rekik A., Allaoui S., Blond E., Gasser A. (2012). Compressive behaviour of dry joints in refractory ceramic masonry. Constr. Build. Mater..

